# Supplementary material for: Comparing the Effect of HPP on the Structure and Stability of Soluble and Membrane-Bound Polyphenol Oxidase from ‘Lijiang Snow’ Peach: Multispectroscopic and Molecular Dynamics Simulation
Source: Foods. 2023 Apr 27;12(9):1820. doi: 10.3390/foods12091820 (PMC10178523; doi:10.3390/foods12091820)
Supplement: Supplementary file 1 [file foods-12-01820-s001.zip › foods-2276872-supplementary.pdf]

Supplement

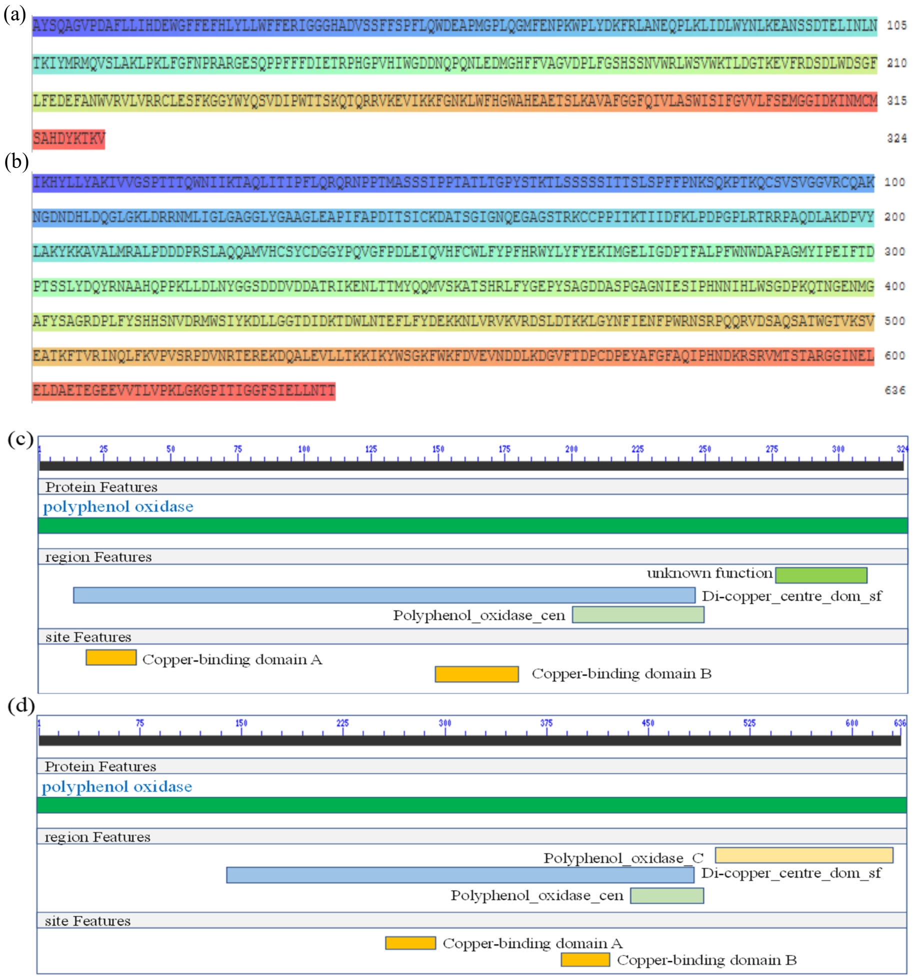

Figure S1. Constitute of amino acid sequence of sPPO and mPPO. The amino acid sequence of sPPO (a) and mPPO (b) obtained from the Entrez protein of NCBI; the regions division according to the functional of sPPO (c) and mPPO (d).

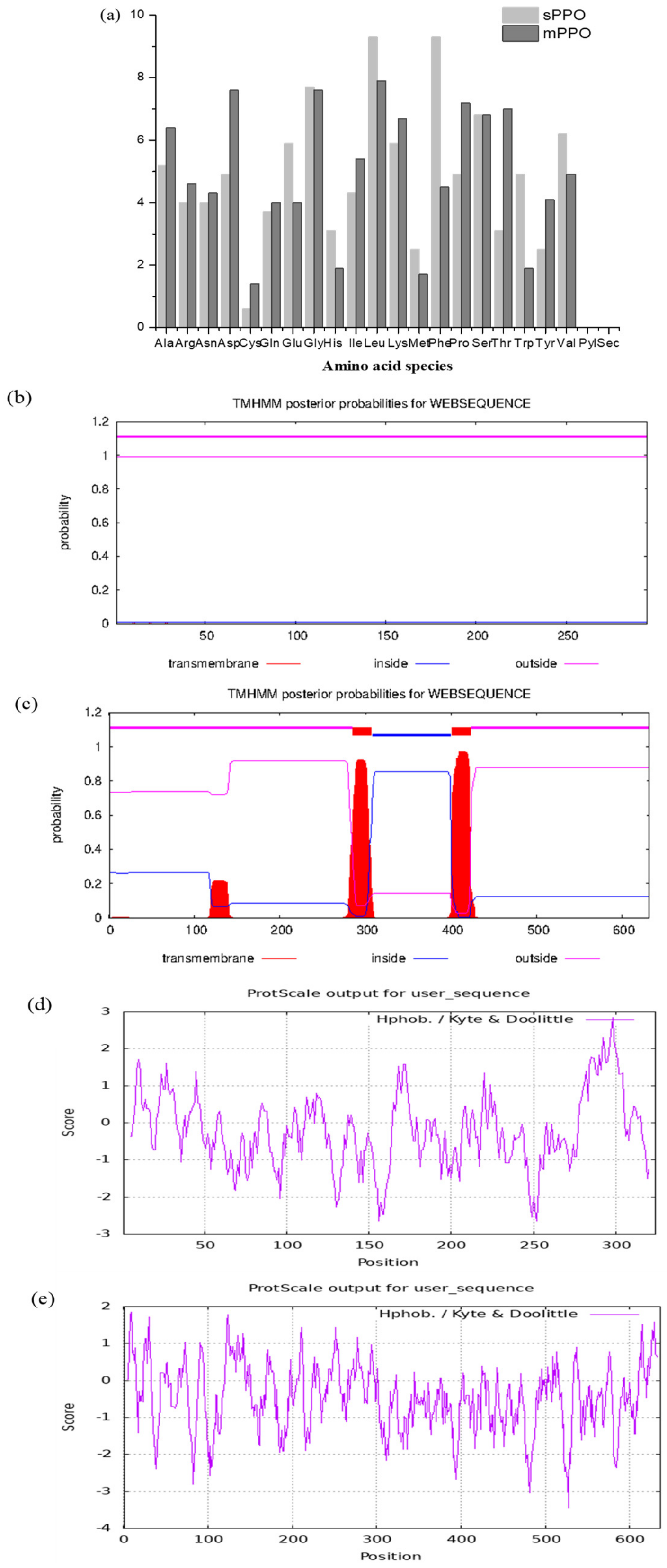

Figure S2. Bioinformatics analysis of sPPO and mPPO. (a) represented the amino acid composition of PPOs; (b) and (c) represented transmembrane region analysis of sPPO and mPPO, respectively; (d) and (e) represented hydrophobicity analysis of sPPO and mPPO, respectively.

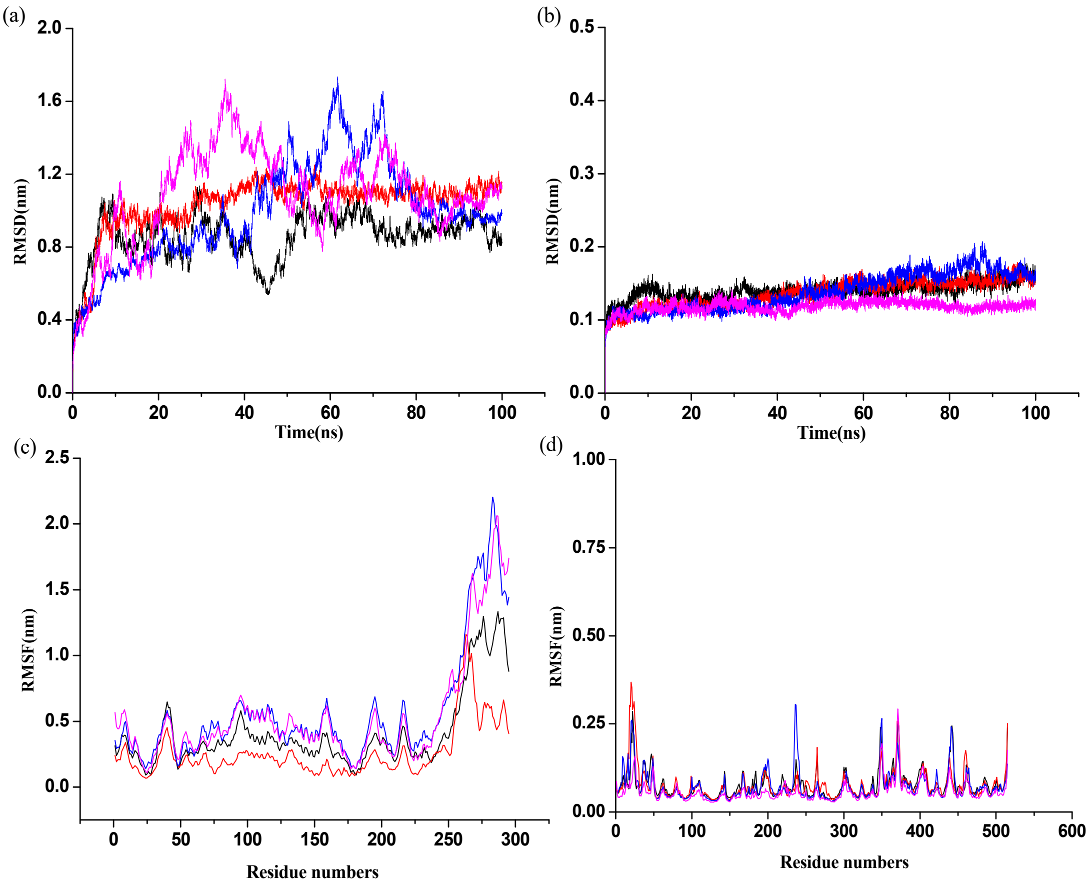

Figure S3. RMSD (a, b) and RMSF (c, d) of sPPO and mPPO simulated by MD simulation under different pressure. (——: 0.1 MPa; ——: 200 MPa; ——:400 MPa; ——: 600 MPa).

Table S1. Physicochemical characteristics and the sub-cellular localization of sPPO and mPPO.

	Molecular formula	Molar extinction coefficient	Instability coefficient	Fat coefficient	Predicted location (s)
sPPO	$C_{1680}H_{2500}N_{448}O_{475}S_{11}$	100045	34.65	76.11	Cytoplasm
mPPO	$C_{3153}H_{4850}N_{846}O_{941}S_{20}$	105240	39.82	72.73	Mitochondrion
